# Immune‐mediated polyneuropathy in cats: Clinical description, electrodiagnostic assessment, and treatment

**DOI:** 10.1111/jvim.16701

**Published:** 2023-05-04

**Authors:** Nicolas Van Caenegem, Léa Arti, Thibaut Troupel, Aurélien Jeandel, Hélène Vandenberghe, Vincent Mayousse, Stella Papageorgiou, Kirsten Gnirs, Stéphane Blot

**Affiliations:** ^1^ Ecole nationale vétérinaire d'Alfort CHUVA, Unité de Neurologie Maisons‐Alfort France; ^2^ Univ Paris Est Créteil INSERM, U955 IMRB “Biology of the Neuromuscular System” Team Maisons‐Alfort France; ^3^ Centre Hospitalier Vétérinaire Advetia Vélizy‐Villacoublay France; ^4^ Centre Hospitalier Vétérinaire Pommery Reims France; ^5^ Highcroft Veterinary Referrals Bristol United‐Kingdom; ^6^ Centre Hospitalier Vétérinaire des Cordeliers Meaux France

**Keywords:** feline, juvenile, nerve conduction, neuromuscular, peripheral nerve, weakness

## Abstract

**Background:**

Suspected immune‐mediated polyneuropathy has been increasingly reported in cats, especially in the last decade, but the condition remains poorly understood.

**Objectives:**

Refine the clinical description and review the classification of this condition based on electrodiagnostic investigation and evaluate the benefit of corticosteroid treatment and L‐carnitine supplementation.

**Animals:**

Fifty‐five cats presented with signs of muscular weakness and electrodiagnostic findings consistent with polyneuropathy of unknown origin.

**Methods:**

Retrospective, multicenter study. Data from the medical records were reviewed. The owners were contacted by phone for follow‐up at the time of the study.

**Results:**

The male‐to‐female ratio was 2.2. The median age of onset was 10 months, with 91% of affected cats being <3 years of age. Fourteen breeds were represented in the study. The electrodiagnostic findings supported purely motor axonal polyneuropathy. Histological findings from nerve biopsies were consistent with immune‐mediated neuropathy in 87% of the tested cats. The overall prognosis for recovery was good to excellent, as all but 1 cat achieved clinical recovery, with 12% having mild sequelae and 28% having multiple episodes during their lifetime. The outcome was similar in cats with no treatment when compared with cats receiving corticosteroids or L‐carnitine supplementation.

**Conclusions and Clinical Importance:**

Immune‐mediated motor axonal polyneuropathy should be considered in young cats with muscle weakness. This condition may be similar to acute motor axonal neuropathy in Guillain‐Barré syndrome patients. Based on our results, diagnostic criteria have been proposed.

**Abbreviations**
ACP: acute canine polyradiculoneuritis
AIDP: acute inflammatory demyelinating polyneuropathy
AMAN: acute motor axonal neuropathy
CHVA: Centre hospitalier vétérinaire Advetia
CHVP: Centre hospitalier vétérinaire Pommery
CIDP: chronic inflammatory demyelinating polyneuropathy
CMAP: compound muscle action potential
CSF: cerebrospinal fluid
ENVA: Ecole nationale vétérinaire d'Alfort
GBS: Guillain‐Barré syndrome
IMPN: immune‐mediated polyneuropathy
IQR: interquartile ranges
MNCV: motor nerve conduction velocity
SNAP: sensory nerve action potential
SNCV: sensory nerve conduction velocity

## 1 INTRODUCTION

Polyneuropathies have been described infrequently in young cats. Several authors have reported non‐hereditary, polyneuropathies, especially in the last decade.^1^, ^2^, ^3^, ^4^, ^5^, ^6^, ^7^, ^8^, ^9^ Although terminologies used in these descriptions are not consistent, they generally refer to the same entity. Initially, breed‐related polyneuropathy was suspected with descriptions mainly in Bengal, but also in Abyssinian, Siberian, and Snowshoe cats.^1^, ^2^, ^3^, ^6^, ^8^ Similar conditions more recently were described in various breeds, including domestic shorthair cats.^4^, ^5^, ^7^, ^9^ Although electrodiagnostic data generally were incomplete, they were consistent with a motor, axonal,^1^, ^2^, ^3^, ^8^ demyelinating,^6^, ^9^ or mixed axonal and demyelinating polyneuropathy.^5^, ^7^ However, involvement of a sensory component has not been evaluated in most published cases.^1^, ^2^, ^8^, ^9^ The origin of the disease is still unknown. Early reports have considered it an idiopathic polyneuropathy,^1^, ^3^, ^5^ whereas more recent reports indicate an immune‐mediated polyneuropathy (IMPN).^2^, ^4^, ^6^, ^8^, ^9^ Various treatment modalities have been described, including corticosteroids, L‐carnitine supplementation, and physical therapy alone. To date, insufficient data is available to allow specific treatment recommendations.

Our primary objective was to refine the classification of this condition based on a large case series with comprehensive electrodiagnostic investigation. In particular, similarities among Guillain‐Barré syndrome (GBS), chronic inflammatory demyelinating polyneuropathy (CIDP), and acute canine polyradiculoneuritis (ACP) are discussed. Our secondary objective was to investigate the potential benefits of corticosteroid treatment and L‐carnitine supplementation in our cohort.

## 2 MATERIALS AND METHODS

### 2.1 Case selection

Medical records at the Ecole nationale vétérinaire d'Alfort (ENVA), Centre hospitalier vétérinaire Advetia (CHVA), and Centre hospitalier vétérinaire Pommery (CHVP) were retrospectively reviewed for cats with muscular weakness and electrodiagnostic findings consistent with polyneuropathy from January 2010 to January 2022. Cats with causes of neuromuscular disease other than idiopathic or IMPN (e.g., diabetes mellitus), incomplete data, notable comorbidities (e.g., trauma), or concurrent central neurological signs were excluded. Data from the medical records included signalment, history, clinical signs, results of electrodiagnostic studies, and follow‐up information. Results of additional diagnostic evaluations, such as CBC, serum biochemistry profile, urinalysis, cerebrospinal fluid (CSF) analysis, infectious disease testing, diagnostic imaging, and muscle and nerve histology were reviewed when available. Of the ENVA cases, 1 has been published previously by 2 of the authors (AJ and SB) and 2 were included in a previous study.^2^, ^4^

### 2.2 Electrodiagnostic studies

Electrodiagnostic studies were performed under general inhalation anesthetic protocol by a board‐certified neurologist or a neurology resident in training supervised by a board‐certified neurologist using similar devices (Nicolet Viking Select or Nicolet Viking Quest; Natus Medical Incorporated, Pleasanton, California, USA) on the left side of the cats.

Electrodiagnostic data were interpreted and compared with retrospectively reviewed results obtained in the intact limb of cats with traumatic plexus injury or non‐neurologic lameness of the contralateral limb from 2010 to 2022.

#### 2.2.1 Electromyography

A disposable bipolar concentric needle electrode (40 mm length, 0.45 mm width, 0.068 mm^2^ sampling area) and a SC ground electrode were used for electromyography. Abnormal spontaneous electromyographic activity (e.g., fibrillation potentials, positive sharp waves, complex repetitive discharges) was graded from 1+ to 3+ (mild to severe) in all cats, according to a published grading scale.^10^

#### 2.2.2 Motor and sensory nerve conduction studies

For the following assessments, polytetrafluoroethylene‐coated stainless‐steel monopolar electrodes of different lengths with 3 mm bare tips were used for stimulation and recording. A ground electrode was placed SC between the stimulation and recording electrodes. At least 3 nerves were tested in all cats. Compound muscle action potentials (CMAP) were obtained with supramaximal stimuli of 0.1 ms duration, delivered at a frequency of 1 Hz. The sciatic‐tibial CMAP was recorded from the plantar interosseous muscle after stimulation of the sciatic notch and hock. The sciatic‐fibular CMAP was recorded from the *tibialis cranialis* muscle after stimulation of the sciatic notch and stifle. Ulnar CMAP was recorded from the palmar interosseous muscle after stimulation of the elbow and carpus. Radial CMAP was recorded from the *extensor carpi radialis* muscle after stimulation of the brachial plexus and distal third of the humerus. Conduction block was defined by >50% reduction of CMAP area when stimulating proximally as compared to distally.^11^ Sensory nerve action potentials (SNAP) were obtained with electrical stimulation applied as a rectangular wave of 0.1 ms duration at a frequency of 5 Hz at supramaximal intensity without motor interference. At least 100 consecutive recordings were averaged for interpretation purposes. The sciatic‐fibular SNAP was recorded from the proximal sciatic‐fibular nerve after SC stimulation of the dorsal part of the paw. Ulnar SNAP was recorded from the proximal ulnar nerve after SC stimulation of the lateral part of the fifth digit. Radial SNAP was recorded from the proximal radial nerve after SC stimulation of the dorsal part of the paw.

#### 2.2.3 Late waves

Monopolar electrodes were positioned similar to distal CMAP recording to record the H‐reflex and F wave. The H‐reflex was obtained for the sciatic‐tibial and ulnar nerves with a submaximal stimulus of 0.1 ms duration. The H‐reflex was identified as the most prominent wave with a submaximal stimulus for the M‐wave or in the absence of an M‐wave. The latency rate was calculated using a previously described equation: limb length/(H latency − M latency).^12^ The F‐wave was obtained for sciatic‐fibular and radial nerves with a supramaximal stimuli of 0.1 ms duration. The F‐ratio was calculated using the equation: (F latency − M latency − 1)/(2 × M latency).^13^

#### 2.2.4 Repetitive nerve stimulation

Repetitive supramaximal stimulation of the sciatic‐fibular nerve was performed with trains of 10 supramaximal stimuli of 0.1 ms duration for each stimulus rate, delivered at different frequencies from 0.1 to 3 Hz. A minimum of 1 minute of recovery time elapsed between trains of stimuli. Compound muscle action potential amplitude and area under the curve were compared among the first, fourth, fifth, and tenth potentials to assess decremental responses.

### 2.3 Muscle and nerve biopsies

Under general inhalation anesthesia, biopsy specimens were collected from the right *triceps brachii*, *biceps femoris*, *cranial tibialis* muscles, and right superficial sciatic‐fibular nerve at the level of the stifle joint as previously described.^14^, ^15^ Cats received analgesia with methadone at 0.3 mg/kg IV and antibiotic prophylaxis with ampicillin‐sulbactam at 20 mg/kg IV.

Muscle biopsy specimens were flash frozen in isopentane precooled in liquid nitrogen (−130°C) and stored at −80°C until further processing. Muscle cryosections (10 μm thick) were stained using standard protocols, including hematoxylin‐eosin, modified Gomori trichrome, ATPase preincubation at different pH (9.4, 4.63, 4.35), periodic acid‐Schiff‐hematoxylin, oil red O, and 2,4‐dinitrophenylhydrazine. All muscle biopsy specimens were analyzed at the time of the study. For each biopsy sample, the degree of myofiber atrophy was evaluated and graded as mild (+), moderate (++), or marked (+++), defined as follows: mild (rare angular myofibers), moderate (multifocal diffuse angular myofibers), and marked (myofibers reduced to pyknotic nuclear clumps or fascicular atrophy). Fiber type groupings and IM nerve branches also were evaluated.

Nerve biopsy specimens were placed on a tongue depressor and immersion fixed in Karnovsky's solution for 2 hours before being transferred to 2.5% glutaraldehyde solution in 0.1 M sodium phosphate buffer (pH 7.4). The specimens then were sent to the Section of Clinical & Comparative Neuropathology, Center for Clinical Veterinary Medicine, Ludwig‐Maximilians‐Universität München, Munich, Germany.

### 2.4 Treatments and follow‐up

All owners were contacted by phone at the time of the study. Recovery status, time from examination to recovery, treatments prescribed, and residual neurological deficits (referred to as sequelae) were reviewed. If multiple episodes were mentioned, only data from the episode for which the electrodiagnostic study was performed (referred to as the studied episode) were used. Time from examination to recovery was defined as the time between the electrodiagnostic study and stabilization of clinical signs (complete recovery or recovery with sequelae). The duration of the studied episode was defined as the sum of the duration of clinical signs at presentation and the time from examination to recovery. If corticosteroid treatment was started later in the absence of improvement with or without another treatment, the cat was considered to be treated with corticosteroids, the time from examination to recovery started from corticosteroid treatment initiation, and the duration of the studied episode included the time from presentation to initiation of corticosteroids.

A cat was considered lost to follow‐up if no data were available regarding clinical examination at least 1 month after the electrodiagnostic study and the owners could not be reached after that time, unless the cat returned to normal during that period.

### 2.5 Statistical description

Because all variables were non‐normally distributed, data are presented as medians and interquartile ranges (IQR). Statistical analysis was performed using R software (version 3.6.3, R Foundation for Statistical Computing, Vienna, Austria) and Excel software (version 2202, Microsoft, USA).

## 3 RESULTS

### 3.1 Clinical presentation

**FIGURE 1:**
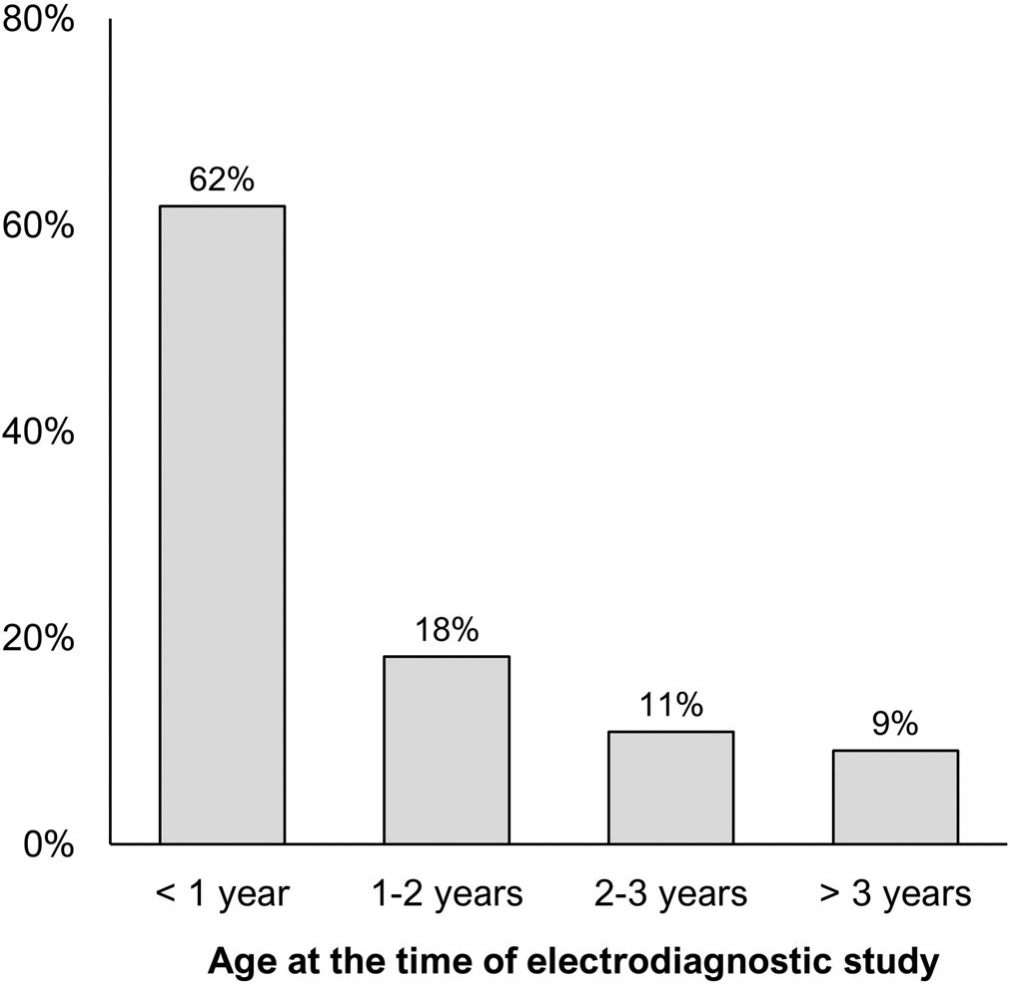
Animal ages at the time of the electrodiagnostic study.

The final cohort included 55 cats: 18 from ENVA, 32 from CHVA, and 5 from CHVP. Of the cohort, 38 (69%) were male and 17 (31%) were female. The male‐to‐female ratio was 2.2. Thirty (55%) cats were neutered. The overall median age at presentation was 10 months (IQR, 7‐20), with 50 cats (91%) < 3 years of age (Figure 1). On examination, the youngest cat was 3 months old, and the oldest cat was 6.5 years old. Fourteen different breeds were represented: mixed breed (n = 18), Birman (n = 9), Bengal (n = 7), Maine Coon (n = 5), Persian (n = 4), British shorthair (n = 3), Siamese (n = 2), Abyssinian (n = 1), Chartreux (n = 1), Devon Rex (n = 1), Norwegian Forest (n = 1), Ragdoll (n = 1), Siberian (n = 1), and Somali (n = 1). Overall median body weight was 3.6 kg (IQR, 3.0‐4.4).

The median duration of clinical signs at presentation was 2 weeks (IQR, 1‐8, data missing for 1 cat). Four cats (7%) had ≥1 similar episodes that resolved without sequelae, with or without treatment. No seasonality was observed; cats were presented in similar proportions in winter (n = 13, 24%), spring (n = 16, 29%), summer (n = 15, 27%), and fall (n = 11, 20%). All cats had pelvic limb weakness associated with difficulty in jumping. Thoracic limbs also were involved in 37 cats (67%), and the owners reported that pelvic limb involvement was either concomitant or occurred before thoracic limb involvement.

Except for neurological dysfunction, physical examination results were unremarkable in all cats. Neurological examination identified postural and gait abnormalities involving all limbs or only the pelvic limbs in all cats, including tetraparesis (n = 37, 67%), paraparesis (n = 18, 33%), plantigrade stance (n = 33, 60%), ataxia (n = 17, 31%), palmigrade stance (n = 5, 9%), and passive cervical ventroflexion (n = 6, 11%). Forty‐seven cats (87%) were ambulatory and 8 cats (13%) were non‐ambulatory. Ambulatory cats generally walked a few steps and then frequently would sit or lie down. Clinical signs were symmetrical. Postural tremors were reported in 2 cats. Postural reactions were decreased in 48 cats (87%), with pelvic limb involvement in 48 cats (87%) and thoracic limb involvement in 39 cats (71%). Decreased or absent spinal reflexes were found in 41 cats (75%), including the pelvic limb withdrawal reflex (n = 41, 75%), patellar reflex (n = 33, 60%), and thoracic limb withdrawal reflex (n = 32, 58%). Bilateral facial paresis was noted in 6 cats (11%). None of the cats had urinary or fecal incontinence.

### 3.2 Clinicopathologic evaluation

Serum biochemical analysis, serum electrolyte concentrations, CBC, urinalysis, and thoracic radiography were performed in 34, 26, 12, 5, and 25 cats, respectively, and were unremarkable in all cats. Serum creatine phosphokinase activity was within the reference range or slightly increased in all tested cats (n = 20; 362 U/L; IQR, 263‐508). Serology for acetylcholine receptor antibody, FeLV, FIV, coronavirus, and *Toxoplasma gondii* testing was performed in 13 cats, and was negative in all cats. Cerebrospinal fluid was collected from the cerebellomedullary cistern in 13 cats with a median duration of 4 weeks of clinical signs before collection (IQR, 2‐10): 11 cats (85%) had total nucleated cell counts and protein concentration within the reference interval, and 2 cats (15%) had increased total protein concentration (0.45 and 1.22 g/L) with total nucleated cell counts within the reference interval (albuminocytological dissociation).

### 3.3 Electrodiagnostic study

#### 3.3.1 Electromyography

Abnormal spontaneous electromyographic activity, including either positive sharp waves and fibrillation potentials (n = 55, 100%) or complex repetitive discharges (n = 2, 4%), was reported with variable intensity from 1+ to 3+ (mild to severe). Pelvic limbs were involved in all cats and thoracic limbs were involved in 52 cats (95%; Table S1). The paraspinal muscles were involved in 8 cats (15%).

#### 3.3.2 Motor and sensory nerve conduction

**FIGURE 2:**
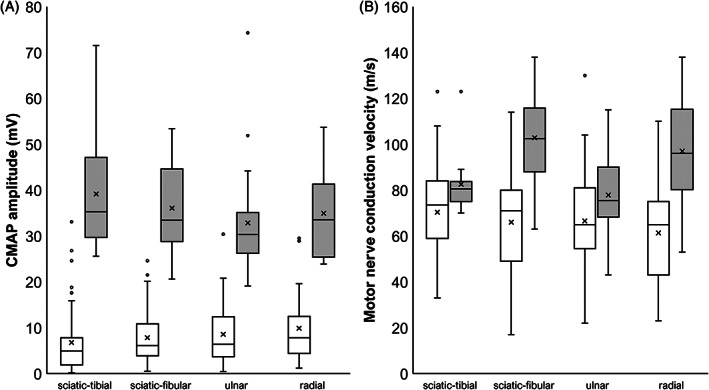
Motor nerve conduction study. (A) Distal compound muscle action potential amplitude for each nerve. (B) Motor nerve conduction velocity for each nerve. Reference values are shown in gray.

All cats had abnormal motor nerve conduction in the tested nerves (Table 1; Figure 2). Polyphasic waves were noted in 22/54 (41%), 14/55 (25%), 8/53 (15%), and 7/33 (21%) cats in the sciatic‐tibial, sciatic‐fibular, ulnar, and radial nerves, respectively. Conduction blocks were found in 15/54 (28%), 9/55 (16%), 22/53 (42%), and 9/33 (27%) cats in the sciatic‐tibial, sciatic‐fibular, ulnar, and radial nerves, respectively. The CMAP amplitude and area were decreased in all cats (Figure 2A). Motor nerve conduction velocities (MNCV) were moderately decreased for at least 2 nerves in 4 cats (7%), with the CMAP amplitude severely decreased. In the other cats (93%), MNCV was within the reference interval or moderately decreased in only 1 nerve (Figure 2B). Sensory nerve conduction was normal in almost all cats (Table 2). In a few cases (7%), sensory nerve conduction velocity was mildly decreased compared to the reference interval, but SNAP amplitude was normal.

**TABLE 1:** Motor nerve conduction for sciatic‐tibial, sciatic‐fibular, ulnar, and radial nerves.

	Amplitude (mV)	Duration (ms)	Area (mVms)	MNCV (m/s)
Sciatic‐tibial (54 affected cats, 14 controls)				
Sciatic notch	2.8 [1.4‐5.6]	6.8 [4.1‐8.8]	3.5 [1.0‐7.2]	74 [59‐84]
	*35.0* [*25.4*‐*42.7*]	*3.2* [*1.3*‐*4.9*]	*19.5* [*14.4*‐*23.0*]	*81* [*75*‐*84*]
Hock	5.0 [1.9‐7.8]	5.8 [3.3‐7.9]	4.5 [2.2‐8.6]	
	*35.3* [*29.7*‐*47.1*]	*3.3* [*1.3*‐*4.3*]	*22.2* [*14.3*‐*23.9*]	
Sciatic‐fibular (55 affected cats, 14 controls)				
Sciatic notch	4.1 [1.7‐7.9]	6.7 [4.6‐10.5]	6.9 [2.8‐15.3]	71 [50‐80]
	*34.0* [*28.4*‐*45.6*]	*7.2* [*4.0*‐*9.9*]	*66.8* [*38.2*‐*88.8*]	*103* [*88*‐*116*]
Stifle	6.1 [3.9‐10.9]	7.8 [4.8‐11.7]	11.9 [7.2‐19.0]	
	*33.5* [*28.8*‐*44.6*]	*5.5* [*3.7*‐*9.2*]	*55.4* [*34.8*‐*68.0*]	
Ulnar (53 affected cats, 22 controls)				
Elbow	2.5 [1.8‐7.4]	5.5 [3.2‐7.7]	4.3 [1.8‐5.9]	65 [55‐81]
	*26.7* [*23.8*‐*28.7*]	*3.7* [*3.0*‐*4.3*]	*19.9* [*16.1*‐*24.2*]	*76* [*68*‐*90*]
Carpus	6.4 [3.7‐12.4]	4.4 [2.6‐7.6]	6.3 [4.3‐11.2]	
	*30.4* [*26.2*‐*35.1*]	*3.5* [*2.9*‐*4.2*]	*20.6* [*16.9*‐*27.3*]	
Radial (33 affected cats, 22 controls)				
Brachial plexus	4.9 [2.2‐10.4]	6.2 [4.3‐8.2]	8.5 [3.5‐12.6]	65 [44‐75]
	*26.8* [*21.4*‐*32.2*]	*5.4* [*3.2*‐*6.8*]	*27.3* [*20.9*‐*30.8*]	*96* [*80*‐*115*]
Elbow	7.8 [4.4‐12.5]	6.6 [3.6‐8.4]	9.8 [8.9‐17.2]	
	*33.6* [*25.4*‐*41.3*]	*5.5* [*2.4*‐*5.9*]	*29.1* [*20.1*‐*43.0*]	

*Note*: The number of tested cats is indicated for each nerve. Reference values are given in italics below the results as medians and interquartile ranges.

Abbreviation: MNCV, motor nerve conduction velocity.

**TABLE 2:** Sensory nerve conduction for sciatic‐fibular, ulnar, and radial nerves.

	Amplitude (μV)	SNCV (m/s)
Sciatic‐fibular (34)	19.5 [10.4‐23.8]	84 [76‐90]
*Controls* (12)	*13.0* [*5.5*‐*23.3*]	*87* [*82*‐*92*]
Ulnar (19)	38.0 [9.9‐49.0]	77 [74‐89]
*Controls* (21)	*37.0* [*20.0*‐*63.0*]	*94* [*75*‐*108*]
Radial (4)	38.0 [17.0‐76.0]	73 [61‐86]
*Controls* (21)	*13.0* [*9.0*‐*20.0*]	*78* [*62*‐*85*]

*Note*: The number of tested cats is indicated for each nerve. Reference values are given in italics below the results as medians and interquartile ranges.

Abbreviation: SNCV, sensory nerve conduction velocity.

#### 3.3.3 Late waves

**FIGURE 3:**
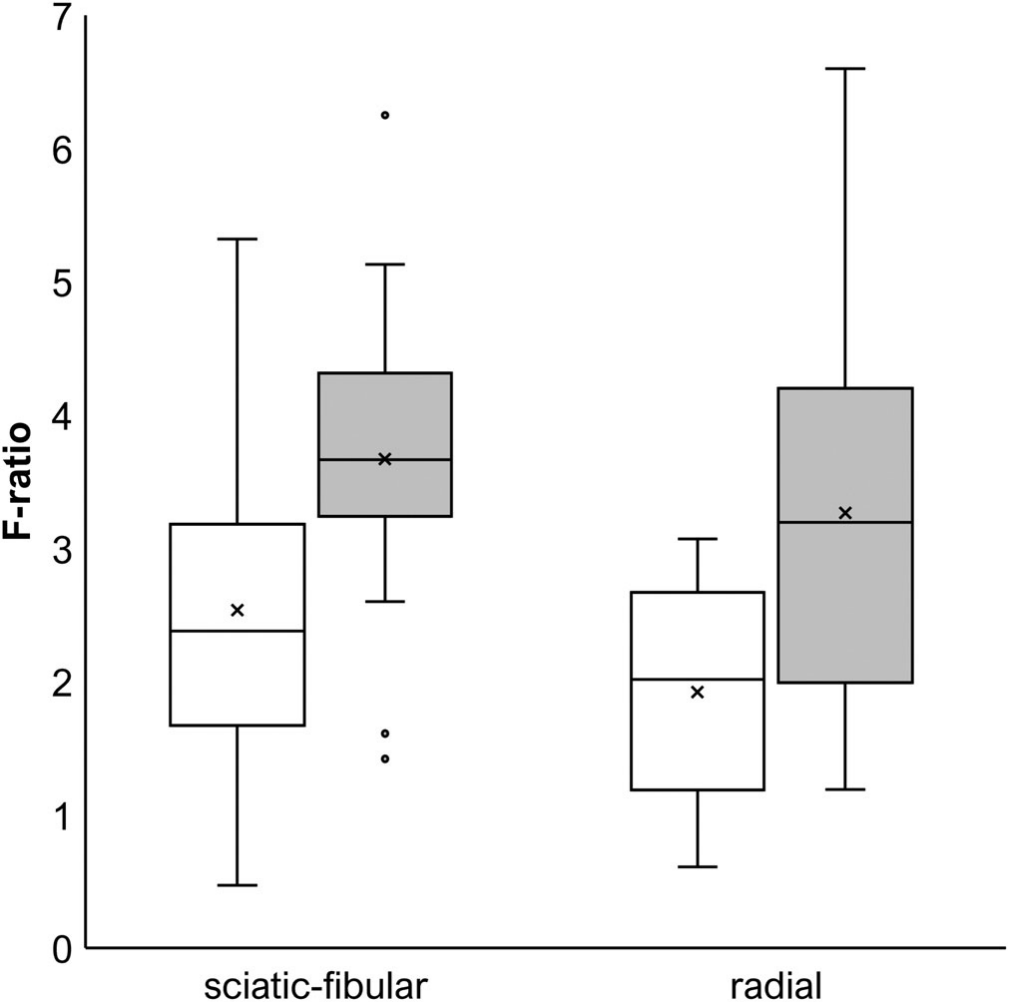
F‐ratio for sciatic‐fibular and radial nerves. Reference values are shown in gray.

The H‐reflex was obtained for the sciatic‐tibial nerve in 42/50 cats (84%) and for the ulnar nerve in 30/47 cats (64%). Because limb size was not available for all cats, latency rate could only be calculated for 8 cats for the sciatic‐tibial nerve and 3 cats for the ulnar nerve. The median latency rate was decreased for both nerves: 23.5 m/s (IQR, 19.5‐28.1; reference [n = 13], 41.8 m/s; IQR, 39.0‐46.1) and 15.4 m/s (IQR, 14.4‐23.6; reference [n = 14], 37.3 m/s; IQR, 33.0‐43.3), respectively. The F‐wave was obtained for the sciatic‐fibular nerve in 37/50 cats (74%) and for the radial nerve in 12/20 cats (60%). The median F‐ratio was moderately decreased for both nerves: 2.38 (IQR, 1.67‐3.18; reference [n = 14], 3.67; IQR, 3.24‐4.32) and 2.02 (IQR, 1.19‐2.67; reference [n = 20], 3.20; IQR, 1.99‐4.2), respectively (Figure 3).

#### 3.3.4 Repetitive nerve stimulation

Only 3 cats were tested. Decremental CMAP amplitudes were observed in 1 cat, with decrements of 21%‐32% and 13%‐29% for amplitude and area under the curve at rate of 0.5 Hz, respectively. In this cat, neostigmine injection did not improve decremental responses and anti‐acetylcholine receptor antibodies titers were within reference interval.

### 3.4 Histopathological evaluation of muscle and nerve biopsies

**FIGURE 4:**
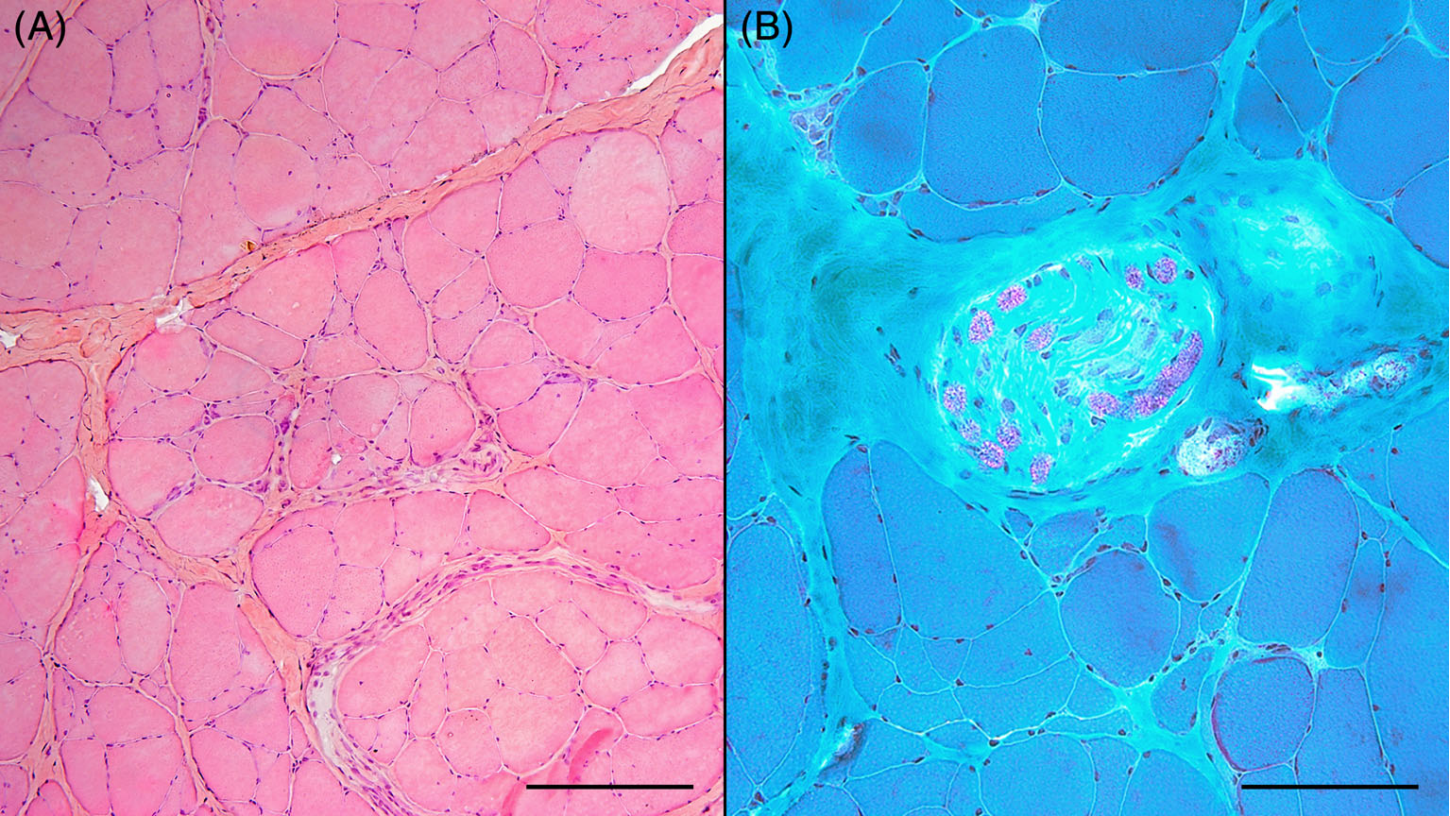
Histochemical evaluation of muscles of the same cat. (A) Diffuse variability in myofiber size without necrosis or inflammatory cells in the *biceps femoralis* muscle (hematoxylin and eosin stain, bar = 200 μm). (B) Intramuscular nerve showing fibers loss and fibrosis in the *tibialis cranialis* muscle (modified Gomori's trichrome stain, bar = 100 μm).

Muscle biopsies were performed in 43 cats (78%), including 42 *biceps femoris*, 18 *tibialis cranial*, and 11 *triceps brachii*. Diffuse variability in muscle fiber size was observed in all muscles, with scattered or clustered atrophic and angular fibers within the same fascicle (Figure 4A). Other abnormalities were rarely observed and, when present, were focal and not prominent. When an IM nerve was present, axons often were depleted: 15/19 in *biceps femoris*, 9/9 in *tibialis cranial*, and 5/5 in *triceps brachii* muscles (Figure 4B). For cases from ENVA, the semi‐quantitative evaluation of the severity of myofiber atrophy is presented in Table 3. Depending on the fascicle, a predominance of type 2 myofibers, type 1 myofibers, or both type 2 and type 1 myofibers was found in 9/18 cats (50%), 2/18 cats (11%), and 1/18 cats (6%), respectively. None of the histological sections showed a grouping pattern. These features were consistent with moderate to pronounced neurogenic myopathy.

**TABLE 3:** Semi‐quantitative evaluation of degree of myofiber atrophy.

	Mild (+)	Moderate (++)	Marked (+++)
*Biceps femoris* (17)	2 (12%)	7 (41%)	8 (47%)
*Tibialis cranialis* (18)	2 (11%)	6 (33%)	10 (56%)
*Triceps brachii* (11)	3 (27%)	4 (36%)	4 (36%)

*Note*: The number of tested cats is indicated for each nerve.

Nerve biopsies were performed in 33 (60%) cats. Two biopsies were nondiagnostic for technical reasons. Among the 31 remaining samples, 27 (87%) were consistent with IMPN with histological evidence of nerve fiber adhesive or invasive inflammatory infiltrates or both directed at the axons, nodes of Ranvier, and Schwann cells,^4^, ^9^ and 2 (6%) were unremarkable. The remaining 2 cats had equivocal findings with an axonal or mixed (axonal and demyelinating), non‐selective, diffuse, chronic neuropathy. These were the 2 oldest nerve biopsy samples in the study (early 2014), which could call into question the final interpretation, considering recent developments in IMPN.^4^

### 3.5 Treatments and follow‐up

After the electrodiagnostic study, 12 cats (22%) received no treatment, 27 (49%) received L‐carnitine supplementation at 50 mg/kg q12h for a median duration of 17 weeks (IQR, 13‐26), and 16 (29%) received corticosteroids for a median duration of 17 weeks (IQR, 9‐26). In 3 cats, corticosteroids were initiated in the absence of substantial improvement after 1 month of L‐carnitine supplementation, and complete recovery was achieved in 5 days for 1 cat and in 3 weeks for 2 cats.

**FIGURE 5:**
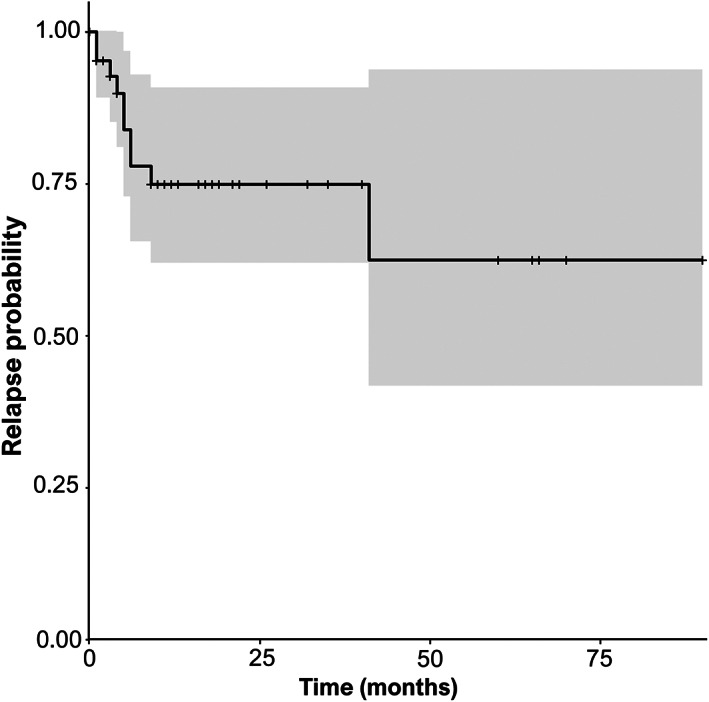
Kaplan‐Meier estimate curve of the risk of developing a relapse.

Follow‐up data were available for 43 cats (78%; Table 4). The median follow‐up duration was 12 months (IQR, 5‐24). All but 1 cat achieved clinical recovery. The median time from examination to recovery was 4 weeks (IQR, 3‐10). By extrapolation, the median duration of the studied episode was 8 weeks (IQR, 4‐15; data missing for 1 cat). Complete recovery was achieved in 37/43 cats (86%). Partial recovery with mild sequelae was reported in 5/43 cats (12%): 3 cats with mild paraparesis and difficulty jumping, 1 cat with limited jumping ability, and 1 cat less agile at play. One cat did not recover after presentation and still was nonambulatory tetraparetic after 2.2 years. The cat was 7 months old at presentation. Relapse was reported in 10 cats (23%): 5 had 1 relapse, 3 had 2 relapses, and 2 had >2 relapses. With the exception of 1 cat with a first relapse 3 years after electrodiagnosis, the first relapse occurred within 9 months of electrodiagnosis in the other cats (Figure 5). Considering the previous episodes in 4 cats, 12/43 cats (28%) had multiple episodes during their lifetime. One cat was euthanized because of multiple relapses despite clinical recovery at each episode, including the studied episode.

**TABLE 4:** Comparison of follow‐up results between the overall sample, and the cats with and without IMPN histological diagnosis.

	Median follow‐up time (months)	Episode duration (weeks)	Relapses	Complete recovery
Overall sample (n = 43)	12 [5‐24]	8 [4‐15]	10 (23%)	37 (86%)
With IMPN histological diagnosis (n = 22)	8 [4‐12]	8 [5‐13]	8 (36%)	19 (86%)
Without IMPN histological diagnosis (n = 21)	19 [12‐35]	7 [4‐18]	2 (10%)	18 (86%)

### 3.6 Analytic study

#### 3.6.1 Comparison between cats with and without IMPN histological diagnosis

The results of motor and sensory nerve conduction studies (not detailed) and follow‐up (Table 4) were similar in the overall cohort and in the cats with and without a nerve biopsy consistent with IMPN.

#### 3.6.2 Relationship between treatment and outcome

**FIGURE 6:**
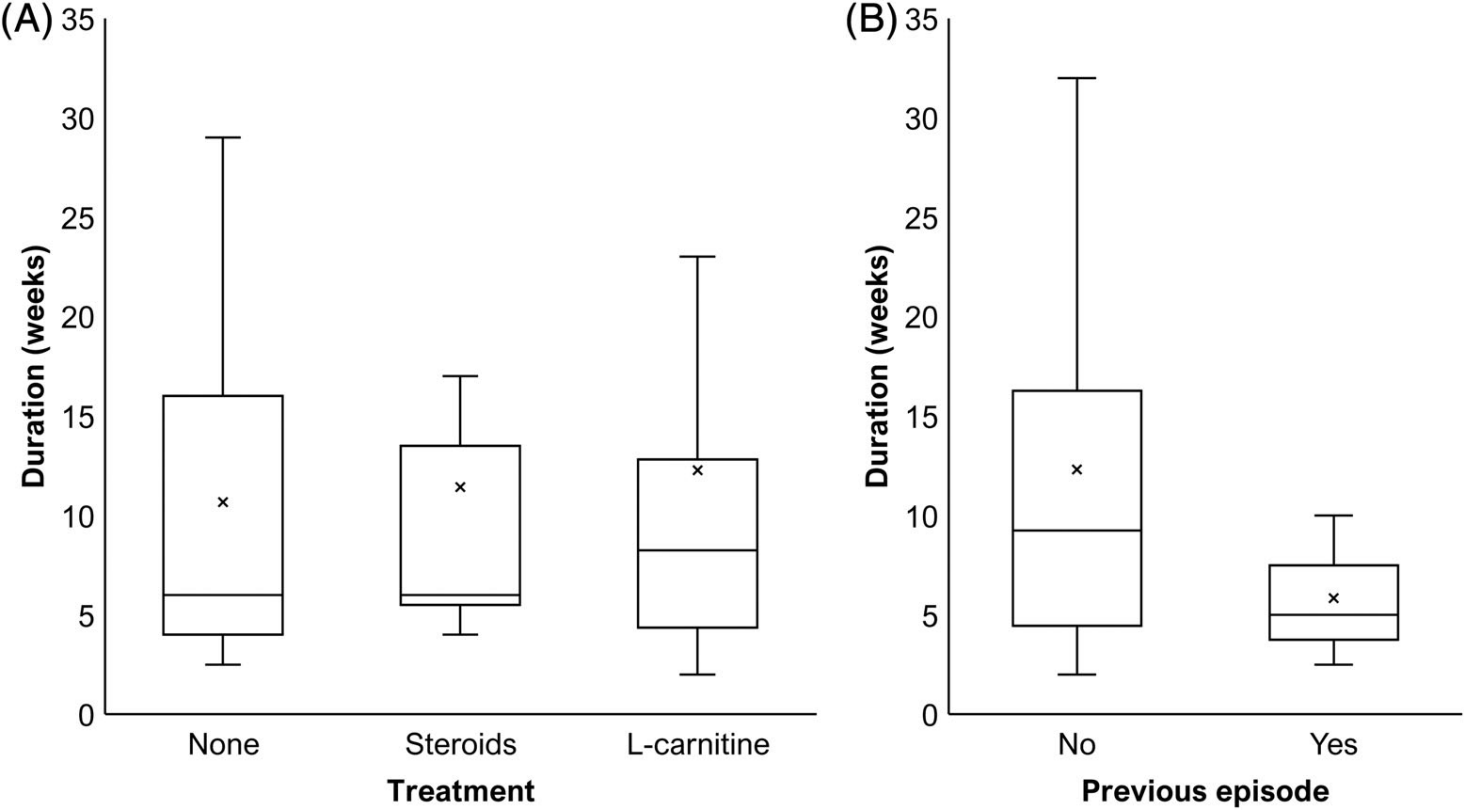
Analysis of duration of the studied episode. Four cats are not included: time from examination to recovery could not be estimated for 3 cats because of sequelae and duration of signs at presentation was missing for 1 cat. (A) Distribution of duration of episodes depending on treatment. The 3 cats receiving corticosteroids in the absence of significant improvement after 1 month with L‐carnitine supplementation were excluded. (B) Distribution of duration of episodes for cats with and without history of previous episodes.

Of the 43 cats with follow‐up, complete recovery was reported in 10/11 (91%) cats receiving no treatment (follow‐up duration, 21 months; IQR, 7‐38), 9/12 cats (75%) that received corticosteroids (follow‐up duration, 14 months; IQR, 4‐23), and 18/20 cats (90%) that received L‐carnitine supplementation (follow‐up duration, 12 months; IQR, 7‐18). The only cat that did not recover was treated with prednisolone (2 mg/kg/day) after presentation. Treatment was gradually discontinued after no signs of improvement. To calculate the time from examination to recovery and the duration of the studied episode, 7 cats were not included: time from examination to recovery could not be estimated for 3 cats because of sequelae or absence of recovery, 3 cats received corticosteroids in the absence of substantial improvement after 1 month of L‐carnitine supplementation, and the duration of signs at presentation was missing for 1 cat. The time from examination to recovery was similar in cats receiving no treatment (n = 11; 3.0 weeks; IQR, 2.5‐9.5), corticosteroids (n = 7; 4.0 weeks; IQR, 2.0‐7.0), or L‐carnitine (n = 18; 4.0 weeks; IQR, 3.5‐10.5). By extrapolation, the duration of the studied episode was similar in cats receiving no treatment (n = 11; 6.0 weeks; IQR, 4.0‐16.0), corticosteroids (n = 7; 6.0 weeks; IQR, 5.5‐13.5), or L‐carnitine (n = 18; 8.3 weeks; IQR, 4.4‐12.8; Figure 6A). Relapses were reported only in treated cats: 3/12 cats (25%) had received corticosteroids and 7/20 cats (35%) had received L‐carnitine supplementation. Of the 3 cats treated with corticosteroids, 2 cats presented with 3 similar episodes that resolved without sequelae before presentation.

#### 3.6.3 Relationship between number of episodes and outcome

Of the 43 cats with follow‐up, the duration of the studied episodes could not be calculated for 4 cats: 3 cats without previous episodes and 1 cat with ≥1 previous episodes. Cats with ≥1 previous episodes showed lower median duration of the episode studied (n = 3; 5.0 weeks; IQR, 3.8‐7.5) compared to cats without previous episode (n = 36; 9.3 weeks; IQR, 4.5‐16.3; Figure 6B).

## 4 DISCUSSION

Our study refines the clinical description of IMPN in cats, with recurrent episodes in young cats, especially males. Similar to the most recent studies, our results do not support a breed‐related disease.^4^, ^7^, ^9^ Some cats had a history of similar episodes that resolved without treatment before presentation. All limbs, or only the pelvic limbs, were symmetrically involved. Electrodiagnostic studies were consistent with motor axonal polyneuropathy, with minimal or no sensory involvement. The overall prognosis with or without treatment was good to excellent, and all but 1 cat achieved clinical recovery. However, our study showed relapse in 28% of cats and sequelae in 12% of cats, which remained mild. The rate of sequelae was lower than noted in previous studies.^6^, ^9^ The time of recovery may be shorter in cats with previous episodes before presentation, but this result should be confirmed in future studies.

Some authors have proposed an analogy between GBS in humans and ACP and IMPN in cats.^4^, ^6^ The diagnostic criteria for GBS are broad, and include progressive muscular weakness in the legs and arms (or only in the legs) and areflexia (or decreased tendon reflexes) in the weak limbs. Additional clinical signs that strongly support the diagnosis include a progressive phase that lasts from days to 4 weeks, relative symmetry, and cranial nerve involvement, especially bilateral facial paralysis.^16^ Except for bilateral facial paralysis in only 11% of the cats, and normal tendon reflexes in weak limbs in a few cats, all criteria were met in our study. We found a predominance of males over females (2.2‐fold). Considering all previously published cases, the ratio of males to females in IMPN affected cats was estimated to be 2.0 (129/63), which is very close to the relative risk of male GBS patients, estimated to be 1.8.^1^, ^3^, ^4^, ^5^, ^6^, ^7^, ^8^, ^9^, ^17^ Similar to GBS patients and ACP affected dogs, treatment with corticosteroids and L‐carnitine supplementation in our cohort did not have any obvious effect on the recovery latency, total duration of the episode, relapse rate, or the sequelae rate compared to the untreated cats.^18^, ^19^ Because 3 cats achieved clinical recovery only after the addition of corticosteroids, we cannot rule out a benefit for a subgroup of cats, although it is possible these cats would have recovered without treatment. No data in the literature support the use of L‐carnitine in these diseases. Similarly, our results did not support its use in cats. Reference treatments currently are IV immunoglobulin therapy and plasma exchange in GBS patients.^16^, ^20^ A recent study found that IV immunoglobulin therapy tended to decrease the time to recover ambulation in dogs with ACP.^21^ This treatment could be investigated in future studies in IMPN‐affected cats, but its value may be limited by the rapid recovery in most cats.

Interestingly, important differences were found in the clinical presentation among IMPN‐affected cats, GBS patients, and ACP‐affected dogs. Guillain‐Barré syndrome typically affects adults and elderly people, with an increased incidence of 20% for every 10 years increase in age.^17^ In dogs with ACP, the trend is similar with a mean age of 7‐8 years.^22^, ^23^, ^24^, ^25^ Conversely, the incidence decreased with age in IMPN‐affected cats: 91% were <3 years old in our study. According to previously published data, the youngest cat was 3.0 months old and the oldest cat was 10.4 years old.^1^, ^3^, ^4^, ^5^, ^6^, ^7^, ^8^, ^9^ Albuminocytological dissociation is another key feature in the diagnosis of GBS.^16^, ^26^ It was found in 64% of GBS patients, with a positive correlation between the number of patients with increased CSF protein concentration and the time from onset of weakness to lumbar collection. After 1 and 2 weeks, >80% and 88% of GBS patients had increased CSF protein concentration, respectively.^26^ Albuminocytological dissociation was found in 43%‐79% of ACP‐affected dogs.^24^, ^25^ In our study, only 15% of affected cats had increased CSF protein concentration despite a late lumbar collection, which is similar to a recent study (15.4%).^9^ Another study reported albuminocytological dissociation in 6/13 (46%) cats.^6^ Considering the low prevalence of albuminocytological dissociation and the absence of abnormal spontaneous electromyographic activity in the paraspinal muscles of most cats, nerve root involvement is questionable. Late wave studies are poorly described in this disease.^1^, ^2^, ^8^, ^9^ The latency rate allows the assessment of the latency of the H‐reflex considering the length of the limbs. The decreased latency rate for the sciatic‐tibial and ulnar nerves is consistent with neuropathy and radiculopathy. The decreased F‐ratio in our cohort compared to that in control cats was consistent with distal nerve involvement. Thus, we suggest that the term motor polyneuropathy might be preferred to polyradiculoneuropathy. Finally, the relapse rate was higher in the IMPN‐affected cats than in the GBS patients. In a recent study, only 5% of GBS patients had a relapse with a median time of 18 weeks between onset of the disease and relapse.^26^ However, approximately 10% of GBS patients had treatment‐related fluctuations defined as disease progression within 2 months after initial treatment‐induced clinical improvement or stabilization.^20^ In IMPN‐affected cats, we cannot exclude that relapses and treatment‐related fluctuations are not confounded.

Classic sensorimotor GBS is the most frequent variant, affecting 30%‐86% of GBS patients. A purely motor variant was reported in 5%‐15% of patients and can occur in patients with acute motor axonal neuropathy (AMAN) or acute inflammatory demyelinating polyneuropathy (AIDP) subtypes.^16^, ^20^ The absence of sensory nerve conduction abnormalities is much more frequent in AMAN patients (94%) than in AIDP patients (15%).^27^ The differentiation between these subtypes is based on their associated previous infections, neurological features, electrodiagnostic results, and serum antibodies. The correct classification of GBS subtypes can be difficult to achieve, especially during the early phase. In a study, 14%‐16% of initial diagnoses were equivocal, and subtype classification changed in 24% of patients at follow‐up, with an increase in the proportion of axonal GBS.^28^ This result may be because of motor nerve conduction slowing and conduction block in the IgG anti‐GM1 AMAN subtype, known as reversible conduction failure (RCF). This feature rapidly may resolve with restoration of conduction velocity and CMAP amplitudes without evidence of temporal dispersion, as would be the case in remyelination.^29^ Moreover, segmental conduction block may be noted in an early phase in AMAN patients. Thus, electrodiagnosis appears to be more reliable between 3 and 6 weeks rather than within the first 2 weeks after GBS onset.^30^ In our study, severely decreased distal CMAP amplitude in ≥2 nerves was consistent with axonal polyneuropathy. In some cats, MNCV was moderately decreased, although it is well known that motor nerves with very low CMAP amplitudes because of axonal degeneration can have moderately decreased MNCV.^31^, ^32^ Electrodiagnosis was performed within a median of 2 weeks after the onset of clinical signs, which may explain the conduction block and decreased MNCV found in some cats. As in GBS, RCF could mimic demyelinating neuropathy in some IMPN‐affected cats.^4^, ^31^ To explain why the AMAN subtype does not fit in the dichotomous classification of demyelinating and axonal neuropathies, it recently has been proposed that this condition is a nodo‐paranodopathy, with RCF indicating nodal and paranodal alterations.^33^ Late infiltrative nodo‐paranodopathy and severe invasion of nodes and paranodes with milder involvement of Schmidt‐Lanterman clefts have been reported recently in IMPN‐affected cats.^4^ Considering all of these results, IMPN in cats has substantial similarity with the AMAN subtype. Etiopathogenesis needs to be refined according to the dosage of anti‐ganglioside antibodies.

In contrast to a recent study^9^ that proposed that IMPN in cats could be similar to juvenile CIDP in humans, our results and those of previous studies did not support this finding. First, according to a previous study, juvenile CIDP differed from the adult form in the absence of sex predilection and motor predominance.^34^ More recent studies challenged these findings with mainly sensorimotor or sensory variants (40/45 patients) and chronic onset (>8 weeks) in the juvenile CIDP population.^35^, ^36^ Purely motor CIDP (only 2% of CIDP patients) was associated with chronic onset and a median age at onset of 48 years.^37^ Second, the classic course of CIDP is a chronic progressive muscular weakness with deterioration continuing for >8 weeks after the onset of the disease. In the previous study, the median time to nadir was 2 weeks.^9^ In 16%‐18% of patients with CIDP, a more rapid weakness with a nadir within 8 weeks was referred as acute onset CIDP. This variant may be difficult to distinguish from fluctuating GBS. In retrospective and prospective studies, acute onset CIDP patients showed significantly more sensory abnormalities and fewer axonal features than GBS patients.^38^, ^39^ Thus, the European Federation of Neurological Societies/Peripheral Nerve Society Guidelines on management of CIDP include “prominent sensory symptoms and signs at presentation” as the main feature for acute onset CIDP in patients, which is the opposite of the motor polyneuropathy found in IMPN‐affected cats.^40^ Third, in the previous study, only 52% of cats had decreased MNCV, and CMAP amplitude was not detailed, making it difficult to interpret the decrease in MNCV.^9^ In CIDP patients, a decrease of MNCV ≥30% below the lower limit of normal (defined as mean − 2 SD) in 2 nerves is required to be considered demyelinating.^40^ In veterinary medicine, it is currently impossible to establish these thresholds in the absence of reliable reference values. Thus, it could be difficult to draw this conclusion with these results regarding demyelinating polyneuropathy. Fourth, as in GBS, albuminocytological dissociation is a major finding in CIDP (93% of patients), which differs from IMPN‐affected cats.^41^ Finally, corticosteroids are superior to no treatment in CIDP patients, which also differs from our results.^35^, ^36^, ^37^, ^40^, ^41^

Our study had some limitations based on its retrospective nature. There was no standardization of clinical data reported by different clinicians. Therefore, some clinical signs may not have been recorded at the time of the clinical examination, and the recorded duration of the episodes may be imprecise. Moreover, because follow‐up by phone does not allow for direct neurological examination, sequelae may be underestimated. Finally, not all of the cats were histologically diagnosed. Nevertheless, the subgroup with nerve histology consistent with IMPN showed similar results to those of the remainder of the cohort for electrodiagnostic and outcome data, except for the relapse rate. Thus, sampling effect cannot be excluded from this feature.

To date, no diagnostic criteria are available for definitive diagnosis. Nerve histology alone is insufficient and may be unremarkable. It should be included in a broader diagnostic approach, based on several criteria. We propose diagnostic criteria in Table 5 adapted from the GBS consensus statement.^20^

**TABLE 5:** Diagnostic criteria for immune‐mediated polyneuropathy in cats, adapted from GBS consensus statement.^20^

	Diagnostic criteria
Features required for the diagnosis	Progressive muscular weakness involving all limbs or just the pelvic limbs with relative symmetry.
Features that strongly support the diagnosis	(1) Young age (juvenile or young adult); (2) History of similar episodes that resolved without treatment; (3) Decreased or absent tendon reflexes in weak limbs; (4) Absence of sensory deficit; (5) Cranial nerve involvement, especially bilateral weakness of facial muscles; (6) Electrodiagnostic examination consistent with motor axonal polyneuropathy; (7) Nerve biopsy with abnormalities consistent with nodo‐paranodopathy.

Abbreviation: GBS, Guillain‐Barré syndrome.

## CONFLICT OF INTEREST DECLARATION

Authors declare no conflict of interest.

## OFF‐LABEL ANTIMICROBIAL DECLARATION

Authors declare no off‐label use of antimicrobials.

## INSTITUTIONAL ANIMAL CARE AND USE COMMITTEE (IACUC) OR OTHER APPROVAL DECLARATION

Authors declare no IACUC or other approval was needed.

## HUMAN ETHICS APPROVAL DECLARATION

Authors declare human ethics approval was not needed for this study.

## Supporting information


**Table S1:** Abnormal spontaneous electromyographic activity in pelvic and thoracic limbs.
