# Supplementary material for: Immune‐mediated polyneuropathy in cats: Clinical description, electrodiagnostic assessment, and treatment
Source: J Vet Intern Med. 2023 May 4;37(3):1088–99. doi: 10.1111/jvim.16701 (PMC10229362; doi:10.1111/jvim.16701)
Supplement: Supplementary file 1 — Table S1: Abnormal spontaneous electromyographic activity in pelvic and thoracic limbs. [file JVIM-37-1088-s001.pdf]

	Number of cats
Plantar interosseous	55 (100%)
Tibialis cranialis	46 (84%)
Gastrocnemius	41 (75%)
Biceps femoris	32 (58%)
Quadriceps femoris	28 (51%)
Semi-tendinous/membranous	25 (45%)
Palmar interosseous	50 (91%)
Extensor carpi radialis	39 (71%)
Flexor carpi ulnaris	39 (81%)
Triceps brachii	33 (60%)
Biceps brachii	33 (60%)
Supraspinatus	20 (36%)
Infraspinatus	18 (33%)

Supplemental Table S1: Abnormal spontaneous electromyographic activity in pelvic and thoracic limbs.
